# Meso-jejunal lymph node dissection has a survival benefit in patients with remnant gastric cancer

**DOI:** 10.1371/journal.pone.0285554

**Published:** 2023-05-10

**Authors:** Sung Eun Oh, Min-Gew Choi, Jun Ho Lee, Tae Sung Sohn, Jae Moon Bae, Ji Yeong An

**Affiliations:** Department of Surgery, Samsung Medical Center, Sungkyunkwan University School of Medicine, Seoul, Korea; Universitá Sapienza di Roma, ITALY

## Abstract

**Background:**

Clinical benefits of the meso-jejunal lymph node (MJLN) dissection in remnant gastric cancer (RGC) patients have not been fully established. Hence, in this retrospective study, we evaluated the survival benefit of MJLN dissection and prognostic significance of MJLN metastasis in RGC patients who underwent gastrojejunostomy reconstruction after their initial gastrectomy.

**Methods:**

We retrospectively reviewed 391 patients who underwent surgery for RGC at our institution between 1996 and 2019. Among them, 60 patients had MJLN dissection. The index value of the survival benefit gained by dissection of the MJLN was calculated by multiplying the frequency of metastasis at the MJLN station and the 5-year overall survival rate (5YOS) of patients with metastasis at that station. When the metastatic rate or 5YOS exceeded 10%, dissection was recommended. An index value of dissection greater than 1.0 was considered significant.

**Results:**

Total metastatic rate of MJLN was 35% (*n =* 21/60). Patients with MJLN metastasis had advanced pathologic stage compared to patients in the no-metastasis group (*p <* 0.001). In T2-T4 RGC patients, the metastatic rate of MJLN was 48.6% (*n =* 17/35), and their 5YOS was 28.4%. The calculated index value was 13.8. Also, patients with MJLN metastasis had a poorer overall survival than those without metastasis. MJLN metastasis was an independent prognostic factor of overall survival in multivariate analysis (HR 6.77, 95%CI 2.21–20.79, *p =* 0.001).

**Conclusion:**

MJLN dissection should be considered for advanced RGC patients who underwent gastrojejunostomy after distal gastrectomy during their initial surgery according to the index value.

## Introduction

Remnant gastric cancer is defined as a malignancy that develops on the remnant gastric stump after partial gastrectomy due to gastric malignant or benign disease [1]. The incidence of remnant gastric cancer after peptic ulcer surgery appears to be decreasing due to improvements in anti-ulcer medications [2]. However, improved screening surveillance with endoscopy and prolongation of the survival of primary gastric cancer patients have increased the incidence of detection of remnant gastric cancer after primary gastric cancer surgery [3, 4].

One of the treatment of remnant gastric cancer is radical surgical resection [5, 6]. However, unlike primary gastric cancer surgery, the optimal range of lymph node dissection for remnant gastric cancer has not been fully established. Japanese guidelines recommend that regional lymph nodes of the stomach not resected during the initial surgery be dissected in advanced remnant gastric cancer. The clinical benefits of splenic hilar lymph node and mesojejunal lymph node dissection have not been established [7]. Interestingly, recent studies that evaluated the patterns of lymph node metastasis in patients with remnant gastric cancer found that meso-jejunal lymph node metastasis was common in patients who had undergone gastrojejunostomy (Billroth II or Roux-en Y) reconstruction during initial surgery [8, 9]. The index value used to predict the therapeutic benefit of dissection of meso-jejunal lymph nodes was 11.0 in patients in the nationwide registry of the Japanese Gastric Cancer Association of patients who had undergone a gastrectomy with gastrojejunostomy due to primary gastric cancer [8].

Adequate lymph node dissection is not defined for patients with remnant gastric cancer. Therefore, in this study, we evaluated the prognostic significance of the index value of meso-jejunal dissection to determine its survival benefits.

## Materials and methods

Between September 1996 and October 2019, 391 patients were diagnosed with remnant gastric cancer (RGC) and underwent surgical treatment at Samsung Medical Center. Among them, those diagnosed with unresectable state of RGC received neoadjuvant treatment. Adjuvant chemotherapy was usually recommended after surgery except for patients with stage T1N0 or T2N0 cancers. The median follow-up period after the second surgery of these patients was 51 months (range 0–294 months).

Clinicopathologic characteristics including age, sex, histology of primary disease, first operation reconstruction method (Billroth I [gastroduodenostomy], Billroth II/Roux-en Y [gastrojejunostomy] or esophagogastrostomy), RGC type, second operation type (completion total gastrectomy, extended completion total gastrectomy, open biopsy or palliative gastrojejunostomy), recurrent tumor curability (R0, R1, or R2), recurrent tumor involvement of greater curvature, No. 10 lymph node dissection, splenectomy, meso-jejunal lymph node dissection, recurrent tumor size, recurrent tumor histology (differentiated or undifferentiated), and recurrent tumor pathologic stage (including depth of invasion, lymph node metastasis, distant metastasis) were ascertained from medical records.

Patients were categorized according to Kaminishi’s classification [10], which defines primary, residual, and recurrent RGCs according to the interval between the first and second operations, primary disease (benign or malignant), and location of the recurrent cancer (anastomosis or non-anastomosis site). Tumor histology was dichotomized as differentiated, which included papillary adenocarcinoma and well or moderately differentiated adenocarcinoma, or undifferentiated, which included poorly or undifferentiated adenocarcinoma, signet ring cell carcinoma, mucinous carcinoma, and other types. Pathologic stage was classified according to the eighth edition of the American Joint Committee on Cancer Classification [11].

The therapeutic value of mesojejunal lymph node dissection was evaluated using an index, which was first proposed by Sasako *et al*. [12]. This index is used to evaluate the therapeutic value of dissection of individual lymph node stations not only in gastric cancer surgery [13] but also in other solid organ cancer surgeries such as pancreatic cancer [14] or colorectal cancer [15]. The survival benefit gained by dissection of meso-jejunal lymph nodes was calculated by multiplying the frequency of metastasis at the meso-jejunal lymph node station and the 5-year overall survival rate of patients with metastasis at that station. When the metastatic rate or 5-year overall survival rate exceeded 10%, dissection was recommended. An index value of dissection greater than 1.0 was considered significant. This cut-off value exceeds the minimum value of the index in regional lymph nodes of primary advanced gastric cancer according to the Japanese Classification of Gastric Carcinomas, 13^th^ Edition [8, 12, 16].

We obtained survival data of the enrolled patients from updated medical records and the National Statistical Office in Korea. Because this research is a retrospective cohort study, the need for ethical approval and consent were waived by the approval from the Institutional Review Board of Samsung Medical Center, Seoul, Korea (SMC 2022-03-020-001).

### Statistical analysis

We used χ^2^ or Fisher exact tests to determine the significance of differences in categorical variables between groups. Five-year overall survival (OS) was calculated by the Kaplan-Meier method. The log-rank test was utilized for univariate analysis. Variables with *p <* 0.05 on univariate analysis were included in multivariate analysis using a Cox proportional hazards model with a backward logistic regression method to identify independent prognostic factors. *P* values < 0.05 were considered statistically significant. Statistical analyses were carried out using SPSS version 27.0 for Windows (SPSS, Chicago, IL).

### Results

Clinicopathologic characteristics of the 391 patients with remnant gastric cancer who underwent surgery are shown in Table 1. At the time of the second operation due to remnant gastric cancer, 59.1% of patients were older than 60 years, and most patients were male (76.5%). Of the 391 patients, 79.3% had malignant primary disease. Reconstruction after primary surgery was mostly performed by gastroduodenostomy (Billroth I, 41.2%) or gastrojejunostomy (Billroth II or Roux-en Y 57.0%). During total gastrectomy, No.10 lymph node dissection was performed in 11.3% of patients, and splenectomy was performed in 23.8%. Among those who had Billroth II or Roux-en Y anastomosis after primary surgery (*n =* 223), meso-jejunal lymph node dissection was performed in 60 patients during their second operation (26.9%).

**Table 1:** Clinicopathologic variables of 391 patients with remnant gastric cancer.

Variables	Number of patients	%
Age, yrs		
≥60	231	59.1
<60	160	40.9
Sex		
Male	299	76.5
Female	92	23.5
Histology of primary disease		
Benign	81	20.7
Malignant	310	79.3
Reconstruction after primary surgery		
Billroth I	161	41.2
Billroth II or Roux-en Y	223	57.0
Esophagogastrostomy	7	1.8
RGC type		
Primary	184	47.1
Residual	129	33.0
Recurrent	78	19.9
Type of surgery		
Completion total gastrectomy	262	67.0
Extended completion total gastrectomy	113	28.9
Open biopsy or bypass	16	4.1
Residual		
R0	347	88.7
R1	14	3.6
R2	30	7.7
Tumor involvement		
Greater curvature (GC)	124	31.7
Not involving the GC	251	64.2
Missing value	16	4.1
No.10 LN dissection		
Yes	44	11.3
No	331	84.7
Missing value	16	4.1
Splenectomy		
Yes	93	23.8
No	282	72.1
Missing value	16	4.1
Meso-jejunal LN dissection		
Yes	60	26.9
No	154	69.1
Missing value	9	4.0
Histology type		
Differentiated	124	31.7
Undifferentiated	243	62.1
Tumor size, cm		
≥4 cm	171	43.7
<4 cm	193	49.4
Missing value		
Depth of invasion		
T1	162	41.4
T2	46	11.8
T3	75	19.2
T4	92	23.5
Missing value	16	4.1
LN metastasis		
N0	276	70.6
N1	44	11.3
N2	37	9.5
N3	18	4.6
Missing value	16	4.1
Pathologic stage		
I	196	50.1
II	83	21.2
III	69	17.6
IV	41	10.5
Missing value	2	0.5

GC: greater curvature

LN: lymph node

RGC: remnant gastric cancer.

Meso-jejunal lymph node metastasis was found in 21 patients (35%), and their clinicopathologic characteristics are listed in Table 2. In patients with meso-jejunal lymph node metastasis, depth of invasion was T3/T4 (*p <* 0.001). High N stage was significantly more frequent in these patients than the other patients (*p <* 0.001), and more advanced pathologic stage (*p <* 0.001) was observed in patients with meso-jejunal lymph node metastasis than in those without meso-jejunal lymph node metastasis.

**Table 2:** Clinicopathologic variables of 60 patients who underwent meso-jejunal lymph node dissection during remnant gastric cancer surgery.

Variables	Meso-jejunal LN metastasis		*p* value^†^
	No (*n =* 39)	Yes (*n =* 21)	
Age, yrs			0.251
≥60	28 (81.8)	12 (57.1)	
<60	11 (28.2)	9 (42.9)	
Sex			0.568
Male	27 (69.2)	16 (76.2)	
Female	12 (30.8)	5 (23.8)	
Histology of primary disease			0.074
Benign	13 (33.3)	12 (57.1)	
Malignant	26 (66.7)	9 (42.9)	
Reconstruction after primary surgery			N/A
Billroth I	0 (0.0)	0 (0.0)	
Bilroth II or Roux-en Y	39 (100.0)	21 (100.0)	
Esophagogastrostomy	0 (0.0)	0 (0.0)	
RGC type			0.164^‡^
Primary	28 (71.8)	17 (81.0)	
Residual	6 (15.4)	0 (0.0)	
Recurrent	5 (12.8)	4 (19.0)	
Type of surgery			0.001
Completion total gastrectomy	30 (76.9)	7 (33.3)	
Extended completion total gastrectomy	9 (23.1)	14 (66.7)	
Residual			0.753^‡^
R0	33 (84.6)	17 (81.0)	
R1	3 (7.7)	1 (4.8)	
R2	3 (7.7)	3 (14.3)	
Tumor involvement			0.645
Greater curvature (GC)	18 (46.2)	11 (52.4)	
Not involving the GC	21 (53.8)	10 (47.6)	
Histology type			0.251
Differentiated	15 (38.5)	5 (23.8)	
Undifferentiated	24 (61.5)	16 (76.2)	
Tumor size, cm			0.088
≥4 cm	24 (64.9)	18 (85.7)	
<4 cm	13 (35.1)	3 (14.3)	
Depth of invasion			< 0.001^‡^
T1	14 (35.9)	0 (0.0)	
T2	5 (12.8)	0 (0.0)	
T3	9 (23.1)	7 (33.3)	
T4	11 (28.2)	14 (66.7)	
LN metastasis			< 0.001^‡^
N0	24 (61.5)	2 (9.5)	
N1	8 (20.5)	3 (14.3)	
N2	5 (12.8)	7 (33.3)	
N3	2 (5.1)	9 (42.9)	
Pathologic stage			< 0.001^‡^
I	17 (43.6)	0 (0.0)	
II	10 (25.6)	3 (14.3)	
III	7 (17.9)	14 (66.7)	
IV	5 (12.8)	4 (19.0)	

^†^Chi-square test or

^‡^Fisher’s exact test.

GC: greater curvature; LN: lymph node; RGC: remnant gastric cancer.

We calculated the index of the estimated survival benefit of meso-jejunal lymph node dissection and our findings are presented in Table 3. Because there were no metastatic nodes in the T1 subgroup, the index value could not be calculated for this group. However, in the T2-T4 group, the index was 13.8. Index value for the T2-T4 subgroups was 28.6 in patients with a benign tumor (primary disease), 16.7 in those with a tumor in the greater curvature (tumor location), and 13.7 in those with an anastomotic tumor location. The index value in the malignant (primary disease) group was 0.0, as none of the six patients with metastatic lymph nodes were alive 5 years after their second operation.

**Table 3:** Index value of the estimated benefit of meso-jejunal lymph node dissection.

	No. of patients who underwent dissection	No. of patients with positive nodes	5YSR of node-positive patients (%)	Index value
T1	14/74 (18.9)	0/14 (0.0)	N/A	N/A
Benign	5/21 (23.8)	0/5 (0.0)	N/A	N/A
Malignant	9/53 (17.0)	0/9 (0.0)	N/A	N/A
GC	5/23 (21.7)	0/5 (0.0)	N/A	N/A
Non-GC	9/51 (17.6)	0/9 (0.0)	N/A	N/A
Anastomosis	8/36 (22.2)	0/8 (0.0)	N/A	N/A
Non-anastomosis	6/38 (15.8)	0/6 (0.0)	N/A	N/A
T2-T4	35/114 (30.7)	17/35 (48.6)	28.4	13.8
Benign	16/43 (37.2)	11/16 (68.8)	41.6	28.6
Malignant	19/71 (26.8)	6/19 (31.6)	0.0	0.0
GC	18/42 (42.9)	9/18 (50.0)	33.3	16.7
Non-GC	17/72 (23.6)	8/17 (47.1)	19.4	9.1
Anastomosis	28/68 (41.2)	14/28 (50.0)	27.3	13.7
Non-anastomosis	7/46 (15.2)	3/7 (42.9)	33.3	14.3

5YSR: 5-year survival rate

GC: greater curvature

Stage IV patients or patients with R1 or R2 resection were excluded.

**Fig 1:**
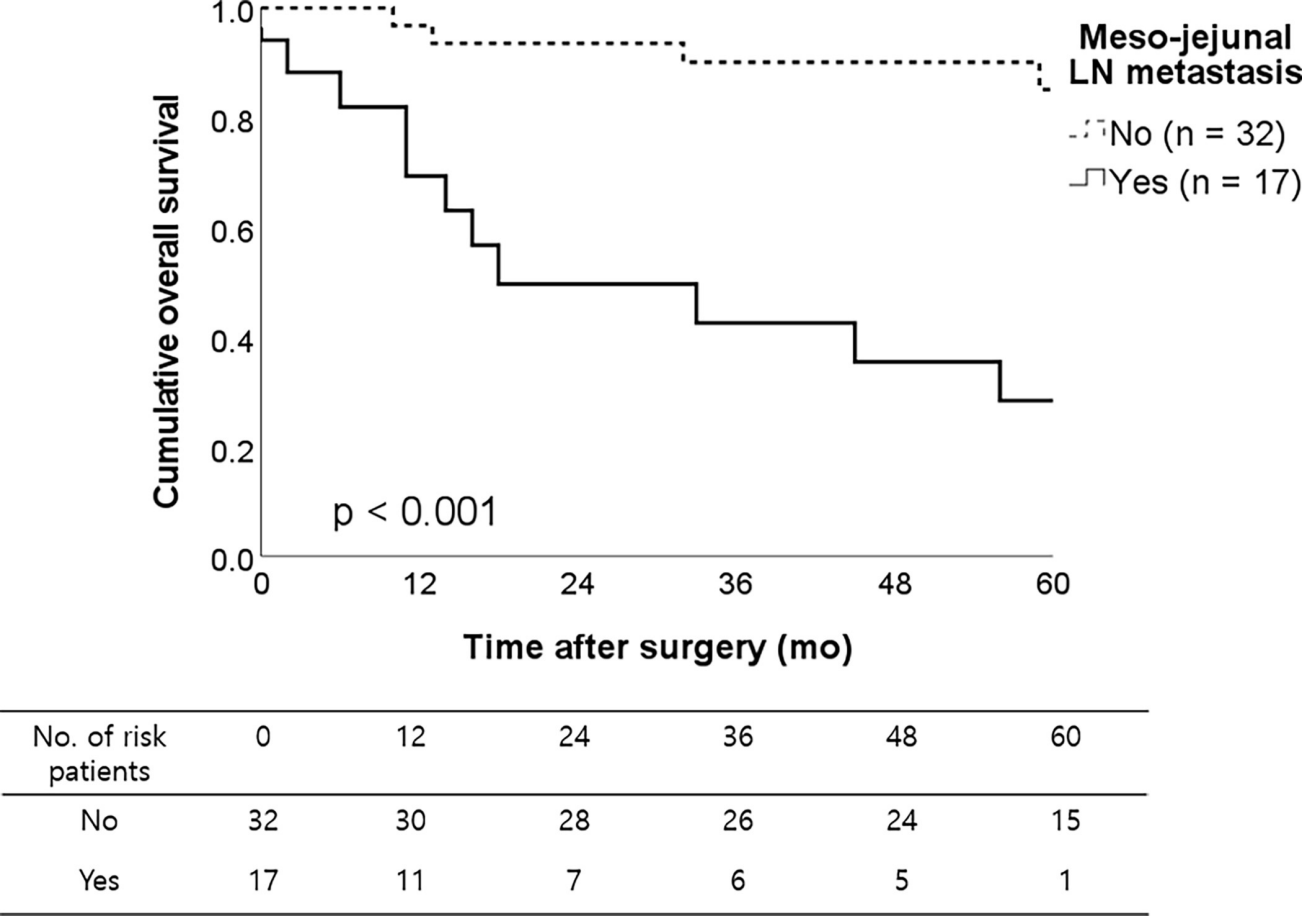
Five-year overall survival rates in patients with meso-jejunal lymph node metastasis and those without metastasis were 28.4% and 85.1%, respectively. The difference between the survival curve was statistically significant (log rank *p <* 0.001). Stage IV patients or patients with R1 or R2 resection were excluded.

Five-year overall survival rates in patients with meso-jejunal lymph node metastasis and those without metastasis were 28.4% and 85.1%, respectively (log rank *p <* 0.001, Fig 1). In multivariate analysis (Table 4), meso-jejunal lymph node metastasis was a significant independent prognostic factor (hazard ratio 6.77, 95% confidence interval 2.21–20.79, *p =* 0.001).

**Table 4:** Univariate and multivariate analyses of the overall survival of patients (*n =* 49) who underwent meso-jejunal lymph node dissection during surgery for remnant gastric cancer.

Variables	Univariate analysis			Multivariate analysis		
	HR	95% CI	*p* value	HR	95% CI	*p* value
Meso-jejunal LN metastasis			<0.001			0.001
No	1.00			1.00		
Yes	7.40	2.49–22.00		6.77	2.21–20.79	
Age, yrs	0.99	0.93–1.05	0.726			
Sex			0.828			
Female	1.00					
Male	1.15	0.33–4.01				
Histology of primary disease			0.688			
Benign	1.00					
Malignant	1.22	0.47–3.15				
RGC type			0.835			
Primary	1.00					
Residual	1.18	0.27–5.27				
Recurrent	1.46	0.41–5.24				
Year of operation			0.017			0.064
Before 2000	1.00			1.00		
2000~2009	0.16	0.03–0.89	0.037	0.15	0.03–0.89	0.037
2010~2019	0.09	0.02–0.47	0.005	0.13	0.02–0.73	0.020
Type of surgery			0.034			0.642
Completion total gastrectomy	1.00			1.00		
Extended completion total gastrectomy	2.82	1.08–7.37		0.74	0.21–2.63	
Tumor involving greater curvature			0.251			
No	1.00					
Yes	0.56	0.51–1.51				
Histology type			0.170			
Differentiated	1.00					
Undifferentiated	2.18	0.72–6.63				
Tumor size, cm	1.11	0.92–1.35	0.290			
Depth of invasion			0.026	N/A (high VIF)		
T1	1.00					
T2	1.89	0.17–21.56	0.610			
T3	4.04	0.81–20.13	0.089			
T4	9.31	1.92–45.18	0.006			
LN metastasis			0.090			
N0	1.00					
N1	2.55	0.63–10.23	0.188			
N2	5.41	1.32–22.11	0.019			
N3	3.83	1.07–13.65	0.039			
Pathologic stage			0.020			0.364
I	1.00			1.00		
II	4.39	0.84–22.96	0.080	2.57	0.42–15.77	0.309
III	8.28	1.83–37.53	0.006	3.44	0.62–19.09	0.157

Stage IV patients or patients with R1 or R2 resection were excluded. CI: confidence interval; HR: hazard ratio; LN: lymph node; RGC: remnant gastric cancer; VIF: variance inflation factor.

## Discussion

Among 391 patients diagnosed with remnant gastric cancer, 57% (223/391) of patients underwent Billroth II or Roux-en Y reconstruction after distal gastrectomy. During their second operation, meso-jejunal lymph node dissection was performed in 26.9% (60/223) and metastasis was found in 35% (21/60) of patients who had undergone lymph node dissection. Patients with metastasis at that station concomitantly had advanced remnant gastric cancer. The index value of the estimated survival benefit of meso-jejunal lymph node dissection was 13.8 in the T2-T4 groups. In addition, meso-jejunal lymph node metastasis was a significant independent prognostic factor in multivariate analysis of overall survival.

One of the treatment of remnant gastric cancer is radical surgical resection [5, 6]. However, optimal surgical treatments have not been fully established, in particular in the field of lymph node dissection [9]. Lymphatic flow and incidence of metastasis after initial gastrectomy for benign disease without lymph node dissection might be the same as for primary gastric cancer. Previous lymph node dissection for initial malignant disease can increase lymphatic flow from the remnant stomach to lymph nodes in the para-aortic, greater curvature, suprapancreatic, jejunal, and colonic mesentery regions [17]. A recent study showed that a different pattern of lymph node metastasis was associated with remnant gastric cancer compared to primary gastric cancer. The pattern of metastasis indicated that jejunal mesenteric lymph nodes should be specifically targeted for *en bloc* resection during completion total gastrectomy [18].

According to previous studies, lymph node involvement in the jejunal mesentery is a phenomenon peculiar to remnant gastric cancer after gastrojejunostomy, with a reported incidence ranging from 7.0% to 46.8% [19]. Due to its high incidence, meso-jejunal lymph nodes should be dissected in remnant gastric cancer patients who underwent gastrojejunostomy during their initial surgery. However, the clinical benefits of this dissection are uncertain, largely because of the poor prognosis of patients with metastasis at this station [7, 19].

In this study, remnant gastric cancer patients with meso-jejunal lymph node metastasis had a significantly worse prognosis than those without meso-jejunal lymph node metastasis. However, the index value suggested that dissection of this station might have therapeutic benefit [8, 12, 16]. The index value was introduced by Sasako *et al* [12]. This index is used to evaluate the therapeutic value of dissection of individual lymph node stations in cancer surgeries [13–15]. Similar results were found in our study. We found that 34.6% (17/49) of patients had meso-jejunal lymph node metastasis and that the 5-year overall survival rate of node positive patients was 28.4%. The calculated index value was 13.8. Thus, we recommend dissection of meso-jejunal lymph nodes in patient diagnosed with advanced (T2-T4) remnant gastric cancer. Metastatic meso-jejunal lymph nodes not detected preoperatively can be dissected intraoperatively and there may be a survival benefit after meso-jejunal lymph node dissection. In a retrospective study using data from the nationwide registry of Japan reported a high index for meso-jeunal lymph nodes in pT2-T4 remnant gastric cancer regardless of the nature of the initial disease (malignant 10.9, benign 17.0) [8]. Interestingly, in our study, the five-year survival of node positive patients who had initial gastrectomy due to malignant disease (*n =* 6) was 0.0.

Limitations of this retrospective study are as follows. First, although the total number of patients who underwent meso-jejunal lymph node dissection for remnant gastric cancer was comparable to that evaluated in other studies [20, 21], this number is still quite low; furthermore, our study was a single center study. To address these shortcomings, external validation of our results and multicenter collaborative data concerning the optimal extent of lymph node dissection, including dissection of the meso-jejunal lymph nodes, is needed. Second, information bias could have occurred when evaluating the medical reports of patients who had their first operation at another institution. Last, this study could not reflect the effect of the development of adjuvant treatment and surgical techniques that might affect patient prognosis during long-term follow-up and the time between surgeries.

In conclusion, the survival benefits of dissection were high for meso-jejunal lymph node dissection in patients with advanced remnant gastric cancer previously anastomosed with gastrojejunostomy. Based on the calculated therapeutic index, dissection of the meso-jejunal lymph node station can be recommended during completion total gastrectomy for advanced remnant gastric cancer. Patients with meso-jejunal lymph node metastasis, however, showed poor survival. Meso-jejunal lymph node may be considered differently from other regional lymph nodes in terms of prognostic significance in patients with remnant gastric cancer. This high-risk group may need thorough follow-up and long-term chemotherapy.

## Supporting information

S1 Data (XLSX)
